# OrthoGarden: a pipeline for propagating phylogenetic trees for nonmodel organisms from short reads and de novo genome assemblies

**DOI:** 10.1093/molbev/msag053

**Published:** 2026-02-27

**Authors:** Jackson H Turner, Ryan D Kuster, Margaret E Staton, John K Moulton

**Affiliations:** Knoxville Department of Entomology and Plant Pathology, University of Tennessee, Knoxville, Tennessee, USA; Knoxville Department of Entomology and Plant Pathology, University of Tennessee, Knoxville, Tennessee, USA; Knoxville Department of Entomology and Plant Pathology, University of Tennessee, Knoxville, Tennessee, USA; Knoxville Department of Entomology and Plant Pathology, University of Tennessee, Knoxville, Tennessee, USA

**Keywords:** evolution, phylogenomics, phylogenetics, orthology, taxonomy, systematics, software, nonmodel

## Abstract

Phylogenomics pipelines are designed to reconstruct evolutionary relationships among groups of organisms. Existing pipelines are dependent upon reference gene sets for which target copies may be retrieved through read-mapping. This read-mapping approach is limited by the availability of reference orthologs closely related to target taxa, which reduces its utility for nonmodel organisms. We introduce OrthoGarden, an automated and containerized de novo assembly-based phylogenomics pipeline aimed to recover accurate and reproducible phylogenies from any combination of short reads and assemblies with particular emphasis on nonmodel taxa. OrthoGarden is tested using 3 datasets of varying size, scope, and taxonomic identity and benchmarked against other phylogenomics pipelines for accuracy. When closely related reference orthologs are available, OrthoGarden produces phylogenies with comparable accuracy to existing pipelines; however, studies limited to distantly related reference orthologs yield increased accuracy using OrthoGarden relative to other mapping approaches. OrthoGarden produces highly accurate phylogenies across a wide range of taxa. Automated phylogenetic reconstruction using genes recovered through all-vs-all orthology inference among selected taxa allows for phylogenomic analysis without requiring in-group reference orthologs. Datasets using nonmodel taxa especially benefit from OrthoGarden's efficacy in the absence of a closely related reference group. Its consistent accuracy, automated usage of computational resources, and ability to utilize both short reads and assemblies make OrthoGarden a community-focused pipeline for both model and nonmodel phylogenomics. OrthoGarden is publicly available at github.com/jacksonhturner/orthogarden.

## Introduction

The study of phylogenetics is essential to understand the relationships between organisms and how they change over evolutionary time scales. The development of phylogenetic reconstructions, or phylogenies, is a widely adopted approach to model evolutionary relationships that strengthens our understanding of speciation, adaptation, gene flow, and other critical aspects of evolutionary comparison (Neafsey et al. 2015; Allen et al. 2018; Lozano-Fernandez 2022; Yang et al. 2023; Dylus et al. 2024). The increasing availability of sequence data provides a wealth of genetic information upon which phylogenies may be created that extends upon the historical use of morphological characteristics (Wiens 2004). Whole-genome sequencing (WGS) data in particular is increasingly abundant due to its applications beyond phylogenetics (Young and Gillung 2020; Lozano-Fernandez 2022). As sequencing technology continuously improves, phylogenetic models must scale accordingly to assimilate unprecedented amounts of genomic sequence data (Young and Gillung 2020). This challenge has been addressed by a multitude of phylogenetic tools that aim to create robust phylogenies from Sanger sequencing products, short (“next-generation”) reads, and long (“third-generation”) reads (Allen et al. 2018; Han et al. 2022; Lozano-Fernandez 2022; Dylus et al. 2024). Phylogenomic pipelines, workflows that leverage existing tools to generate evolutionary reconstructions from WGS data, are employed for this purpose (Allen et al. 2018; Dylus et al. 2024; Gupta et al. 2025). Many of these pipelines are fully automated, allowing for continuous progress without the need for user intervention (Dylus et al. 2024; Gupta et al. 2025). As each phylogenomic pipeline carries its own assumptions and optimal data types, identifying the most appropriate pipeline for a given set of sequencing data can be a challenging task.

Conceptual approaches to phylogenomic pipelines differ in their methods in using single copy exons (orthologs). The proper selection of loci is fundamental to achieving the correct topology for phylogenies, as introducing loci with divergent evolutionary histories can produce misleading results (Young and Gillung 2020; Lozano-Fernandez 2022). A traditional approach to phylogenomics relies upon including only orthologous loci (usually exons), which may be chosen from a curated database (Allen et al. 2018; Kriventseva et al. 2019; Dylus et al. 2024) or identified de novo. These orthologous loci may be acquired from whole genome alignments, comparison with a reference genome, or through orthology inference of annotated loci (Emms and Kelly 2019). An alternate approach forgoes the use of orthologous exons to include homologous or multi-copy loci, such as introns, ultraconserved elements, or c-genes (Gupta et al. 2025). These approaches have been used to produce well-supported phylogenies (Yang et al. 2023; Dylus et al. 2024; Gupta et al. 2025) but rely on high-quality genome assemblies, thus orthology remains widely used in phylogenomics (Young and Gillung 2020; Lozano-Fernandez 2022; Stiller et al. 2024).

To recover orthologs from WGS data, 2 main approaches for selecting orthologous exons are employed. One approach begins with read-mapping to a reference genome or set of genes, followed by local de novo assemblies for all reads mapping to each reference gene (from here referred to as read-mapping approach). Recent pipelines utilizing a read-mapping approach include aTRAM and read2tree (Allen et al. 2018; Dylus et al. 2024). A second approach assembles all reads first, followed by de novo gene or exon annotation and ortholog identification from the resulting, often highly fragmented, contigs (from here referred to as assembly approach) (Allen et al. 2018; Montoliu-Nerin et al. 2021; Yang et al. 2023; Dylus et al. 2024). Either approach can unintentionally introduce nonorthologous genes into a dataset, obfuscating phylogenetic signal by including loci with divergent evolutionary histories (Lozano-Fernandez 2022; Fleming et al. 2023).

Read-mapping approaches that rely on a selected reference gene set may introduce unintentional error in multiple ways. Curated reference ortholog sets such as those from OMA (Orthologous Matrix) and OrthoDB (Kriventseva et al. 2019) can introduce error if an evolutionarily distant reference organism is selected, due to high sequence divergence, gene loss, and/or presence of gene copies (paralogs) (Lozano-Fernandez 2022). After selection of a reference gene set, read-mapping pipelines may reconstruct nonorthologous genes for the target taxa. This may occur when paralogs are present and their reads map equally well to a single reference gene or when a gene has been lost in a lineage and reads from a homolog map to the reference gene. These problems can be further exacerbated by low sequence coverage, which may have insufficient reads from orthologous genes for high-quality reconstruction. Reads with high sequencing depth (>100×) elevate the likelihood for inaccurate de novo assembly of target loci by introducing a greater number of nontarget reads, disrupting phylogenetic signal (Dylus et al. 2024). Deeply sequenced reads may allow for chimeric reconstruction of paralogous consensus sequences leading to incorrect inference of relationships between taxa. Downsampling of highly sequenced taxa may ameliorate this issue but is currently not implemented in existing phylogenomics pipelines.

The vast majority of taxonomic groups lack high-quality reference genomes or ortholog resources from closely related taxa, suggesting a read-mapping approach may be less accurate for nonmodel species. Assembly-based pipelines avoid the bias of reference genes (Lozano-Fernandez 2022). This allows for increased accuracy in identifying orthologs, provided the sequence data for each target taxa is deep enough to produce assemblies with sufficient contiguity for annotation (Montoliu-Nerin et al. 2021; Yang et al. 2023). Higher read depth improves the completeness of de novo whole genome assembly allowing for a more informed comparison of loci between taxa for orthology inference (Dominguez Del Angel et al. 2018). Orthology inference, wherein loci are evaluated for their ability to best represent the evolution within a taxonomic group, is contingent upon identifying a set of exons that are complete for all or most target taxa for accurate results (Young and Gillung 2020; Montoliu-Nerin et al. 2021; Lozano-Fernandez 2022). While potentially more accurate, assembly-based pipelines may impose a high computational demand. All-vs-all orthology inference compares available genes among all included taxa, and the high number of comparisons between loci make it a resource-intensive process (Young and Gillung 2020). Taxonomic groups such as plants or fungi may contain repetitive elements and high numbers of paralogous genes which further increase the computational burden of orthology inference (Zuntini et al. 2024). De novo whole genome assembly and annotation are also demanding processes that require extensive processing time and computing power (Dominguez Del Angel et al. 2018).

No standardized pipeline for a reference-free ortholog selection from raw reads and assemblies yet exists (Steenwyk et al. 2023), leading to a community need for an easy-to-use, automated, orthology-based phylogenomics pipeline applicable to nonmodel taxa. Existing phylogenomics workflows are composed of separate individual processes such as loci capture, orthology inference, alignment, masking, and tree inference where data must be manually passed between steps (Johnson et al. 2018; Shen et al. 2018; Yang et al. 2023). Automating the flow of hundreds of samples across these existing processes into a cohesive pipeline would reduce analysis time and user input. Additionally, several existing pipelines accept only short read sequencing data sets for each taxon as input (Allen et al. 2018; Dylus et al. 2024). Applying all-vs-all orthology inference between in-group taxa as in OrthoFinder (Emms and Kelly 2019) would allow both short reads and assemblies as inputs, integrating the many genome assemblies publicly available through NCBI in addition to short read inputs.

Here, we introduce OrthoGarden, an automated, scalable, assembly-based phylogenomics pipeline for model and nonmodel eukaryotic taxa. OrthoGarden is a novel phylogenomics pipeline that occupies a niche among existing tools by not requiring a set of reference genes, leveraging both reads and assemblies as inputs, functioning well with reads at high sequencing depths, filtering genes through orthology inference, and being fully automated and easy to implement. We benchmark OrthoGarden's phylogenetic reconstruction accuracy with comparable pipelines using 3 published datasets. OrthoGarden's unique suite of features provides it particular utility in supporting intrafamily systematics without the need for high-quality assemblies or reference genes. To model its anticipated use-cases with nonmodel taxa, we assess its improvement over a read-mapping approach that would include the influence of reference gene selection.

## Results

The mosquito dataset chosen for benchmarking represents a well-studied reference organism (*Anopheles gambiae*) with a well-established phylogeny. Different reference genes sets were chosen for the read-mapping pipelines read2tree and aTRAM to simulate scenarios where closely related reference genes are or are not available. A reference phylogeny generated from 21 selected taxa (Neafsey et al. 2015) was used to benchmark reconstructions for accuracy. OrthoGarden, aTRAM with an *Anopheles* reference gene set, and ROADIES (Gupta et al. 2025) reproduced the reference phylogeny with the most accuracy with a Jaccard Robinson-Foulds (JRFD) score of 3. Read2tree with a close reference gene set was slightly less congruent, and comparable to aTRAM with a distant reference gene set from *Drosophila* (JRFD = 6; Fig. 2h). Read2tree results with a distant gene set from *Drosophila* (Fig. 2d) or a distant gene set from Diptera (excluding mosquito orthologs; Fig. 2e) were both notably less congruent with a JRFD of 12 for each. This demonstrates that genes acquired through read-mapping were strongly impacted by choice of reference genes (Fig. 2d, g, and e). Reference gene sets derived from *Drosophila* or Diptera increase the branch lengths of taxa in the *A. gambiae* species complex and deteriorate accurate relationships between in-group clades. The phylogeny produced with aTRAM using an *Anopheles* reference was identical to Neafsey et al. (2015), but one substituting the closest evolutionary reference with a more distant organism in *Drosophila* demonstrated discordance (Fig. 2a). Notably, when using close evolutionary proxies that excluded Culicidae, read2tree recovered relatively inaccurate topologies and paraphyly within major groupings (Fig. 2f and h). OrthoGarden produced phylogenies with topologies identical to Neafsey et al. (2015) irrespective of the selected Augustus reference organism (Fig. 2a–c).

**Figure 1:**
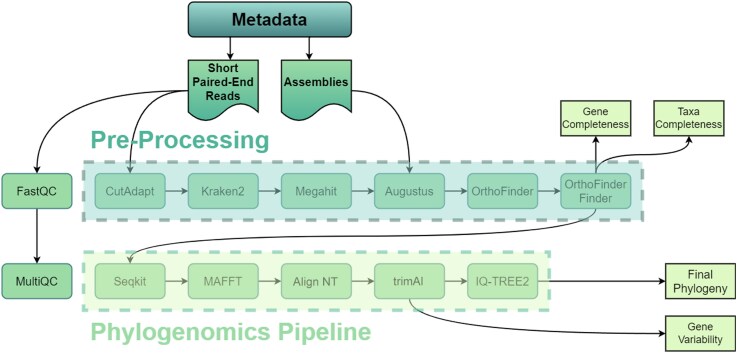
Overview of OrthoGarden pipeline.

**Figure 2:**
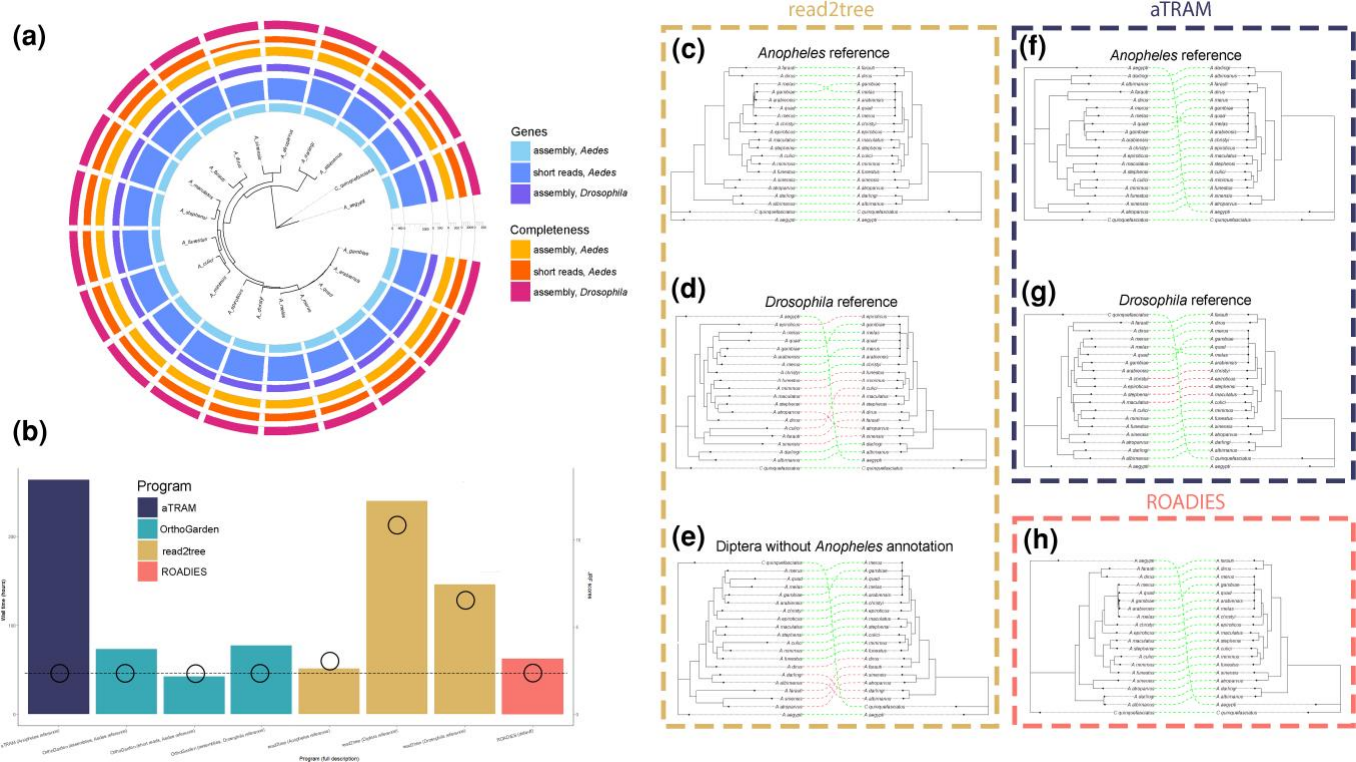
Concordance and performance benchmarking across methods using the 21 taxa mosquito dataset. a) Running OrthoGarden with short read inputs using both *Aedes* and *Drosophila* annotation and assembly inputs produced an identical topology, which is displayed. Tips display bar plots demonstrating the numbers of genes recovered from each method and the number of genes at least 50% complete. b) Run times (barplots) and JRFD scores (open circles) are displayed for the benchmarking results across tools. Cophylogenies comparing topologies produced from c–e: read2tree, f and g: aTRAM, and h) ROADIES are compared against a reference tree (Neafsey et al. 2015). Dashed lines in cophylogenies connect like taxa across trees; green lines represent concordant topologies of like taxa and red lines show discordance.

Orthogarden was further tested with 2 input formats (fasta vs fastq) and 2 Augustus training models (“mosquito” vs “fly”). All tests yield identical JRFD values of 3. This benchmarking demonstrates OrthoGarden's ability to reconstruct accurate relationships by inferring orthology using an all-to-all comparison.

**Figure 3:**
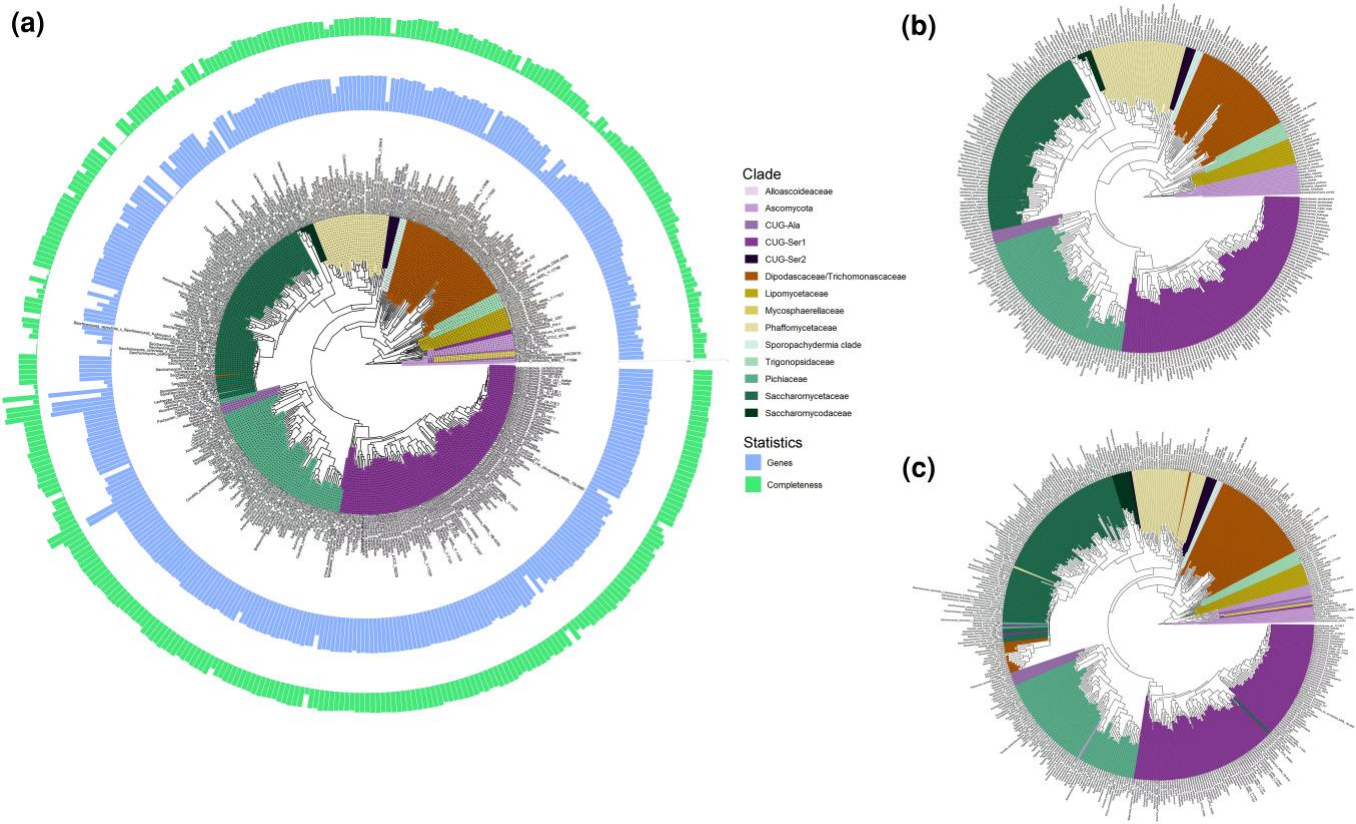
OrthoGarden produces reconstruction comparable to state-of-the-art phylogenomics pipelines for a 410-taxa yeast dataset. a) Phylogenetic reconstruction using OrthoGarden. The number of recovered genes and genes at least 50% complete are reported per taxon. b) Phylogenetic reconstruction using read2tree. a) Reference tree from Shen et al. (2018). Major taxonomic groupings of terminal taxa are colored from clades provided in Shen *et al*. for visual comparison between methods.

A large yeast dataset was selected to evaluate OrthoGarden's performance given an abundance of sequence information, which may pose a computational challenge for tree and orthology inference. A reference phylogeny constructed from selected taxa using filtered single copy orthologs from genome assemblies (Shen et al. 2018) alongside one recovered from a read-mapping alternative (Dylus et al. 2024) were used to benchmark for accuracy. Neither read2tree nor OrthoGarden achieved a topology entirely concordant with the reference (Fig. 3); however, conserved taxonomic groupings were recovered by both. Overall, OrthoGarden produced a more similar topology than read2tree. Consistent paraphyly is observed in both read2tree and OrthoGarden within the Ascomycota, Phaffomycetaceae, Pichiaceae, and Saccharomycetaceae clades. Notably, a subclade of *Wickerhamiella* species is nested within the Saccharomycetae superclade in the read2tree phylogeny that is not present in the OrthoGarden or reference trees. These divergent topologies may be due to the inclusion of additional taxa to those present in the reference tree (Dylus et al. 2024) or incorrect identification of taxa on NCBI which may modify the topologies of these trees, especially out-group taxa. The inclusion of paralogous genes in the original reference tree may further contribute to discordant topologies observed here (Tice et al. 2021). These results demonstrate OrthoGarden's efficacy in reproducing a phylogeny from a large dataset comparable to reconstructions generated from state-of-the-art phylogenomics pipelines.

**Figure 4:**
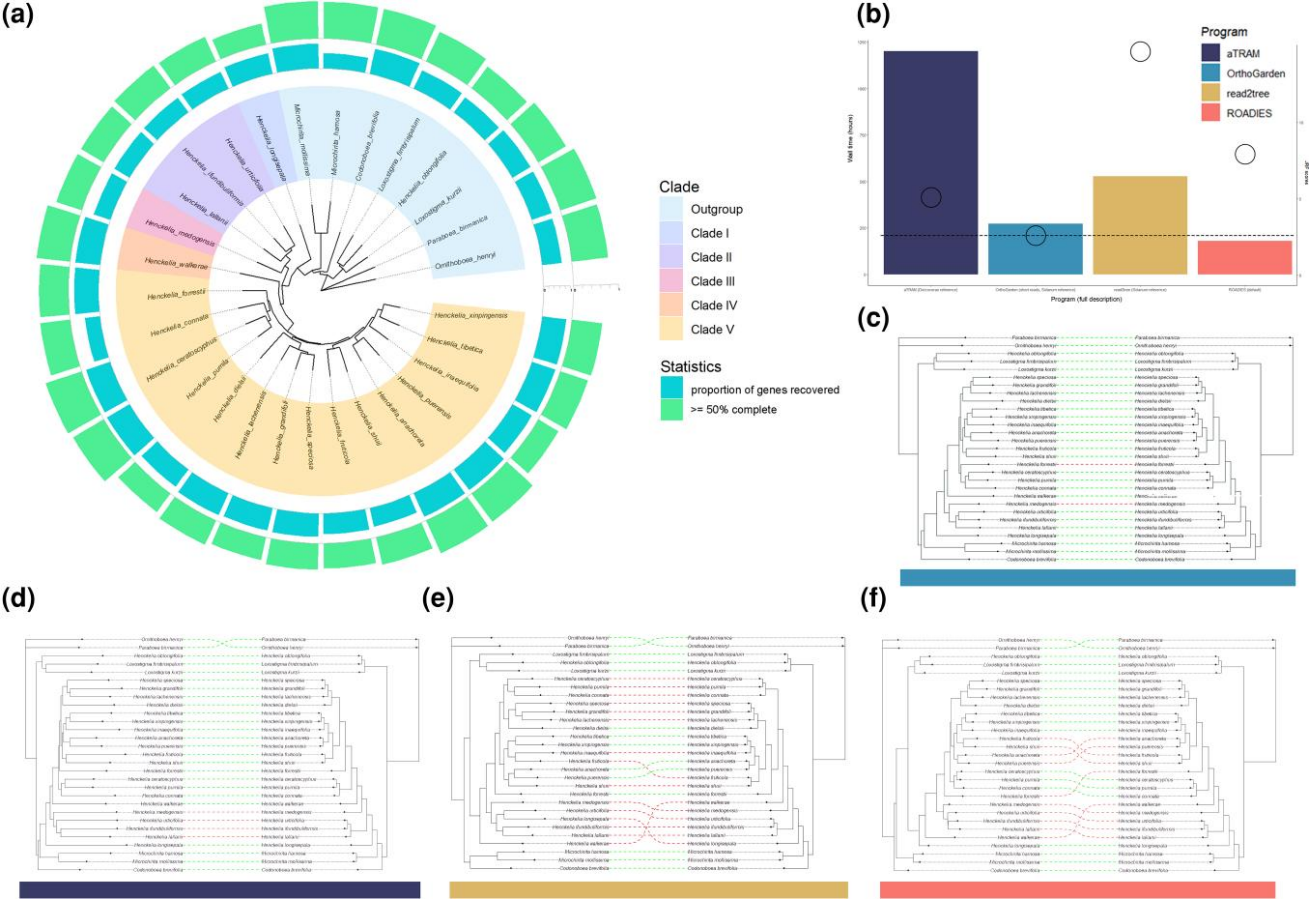
Concordance and performance benchmarking across methods using the 29 taxa *Henckelia* dataset. a) A phylogeny produced by OrthoGarden; the proportion of recovered genes and the number of genes that are at least 50% complete are displayed for each taxon. b) Runtime and concordance benchmarking of 4 different phylogenomics pipelines (aTRAM, OrthoGarden, read2tree, and ROADIES) is shown, with run times (barplots) and JRFD scores (open circles) reported for each. The corresponding cophylogenies (indicated by solid color bar) were constructed using OrthoGarden (c), read2tree (d), aTRAM (e), and ROADIES (f) and were compared with a reference phylogeny (Yang et al. 2023). Dashed lines of cophylogenies connect like taxa across trees; green lines represent concordant topologies of like taxa and red lines show discordance.

A plant dataset within Gesneriaceae with particular focus on *Henckelia* was chosen for benchmarking due to its status as a nonmodel taxonomic group, as it exhibits a high evolutionary distance from common reference organisms (Yang et al. 2023). An extensive history of hybridization and polyploidy in Gesneriaceae provide an opportunity to evaluate OrthoGarden's ability to address the integration of duplicated genetic content typical in some plants and fungi for accurate phylogenetic reconstruction (Yang et al. 2023). Monophyly of *Henckelia*, except for *H. oblongifolia*, a known taxonomic outlier, was recovered across all benchmarked pipelines, consistent with a robust phylogenomic reconstruction from a previous study (Cai et al. 2019; Yang et al. 2023). Internodes anchoring the topology of out-group taxa and nodes depicting interclade relationships were the most variable across methods (Fig. 4c–f). The reconstructions recovered from the aTRAM and ROADIES pipelines performed here produced topologies similar to the reference phylogeny but with divergent subclade relationships (JRFD = 5 and 7.23, respectively). Read2tree produced the reconstruction with the least concordant topology to the selected reference, with considerable clade shifts in both in-group and out-group taxa (JRFD = 17). Of benchmarked pipelines, the reconstruction generated with OrthoGarden exhibits the topology most concordant with the chosen reference tree (JRFD = 3). Results from this dataset demonstrate OrthoGarden's ability to generate accurate phylogenies from taxa with potentially challenging genomic features.

With access to high performance computational resources, OrthoGarden demonstrates run times comparable to and often surpassing other phylogenomics pipelines while producing final topologies highly concordant with published phylogenies in tested benchmarking datasets. Run times for OrthoGarden most closely resembled those for ROADIES and read2tree (using a closely related reference organism) across benchmarking datasets (Figs 2b and 4b). Notably, OrthoGarden demonstrated a shorter run time for short read inputs than assembly inputs (Fig. 2b). OrthoGarden, despite being an assembly-based pipeline, produces accurate final topologies using short reads with at least 3× coverage (Figure S15).

## Discussion

Our benchmarking results demonstrate that the assembly- and orthology-based pipeline, OrthoGarden, produces accurate phylogenies irrespective of evolutionary distance of in-group taxa to a model organism. Across the 3 datasets used for benchmarking, OrthoGarden produced phylogenetic reconstructions with accuracy comparable to or greater than those observed with other pipelines. Benchmarking comparisons of the mosquito dataset demonstrate highly congruent topologies produced across all methods with deteriorating accuracy in read2tree with the use of a genetically distant reference organism. OrthoGarden produced a more accurate phylogeny of 416 yeast accessions than read2tree with all major clades congruent to those exhibited in the reference phylogeny. OrthoGarden was also the most accurate method used in the plant dataset, with read2tree, aTRAM, and ROADIES producing relatively incongruent phylogenies. The accuracy of read2tree phylogenies may be reduced by the removal of OMA taxa from final phylogenies and the relatively low number (200) of genes used. Phylogenetic reconstructions produced by OrthoGarden are observed to be consistent regardless of assembly or paired short-read inputs.

Our results demonstrate that read-mapping approaches recover accurate topologies when provided ortholog sets from a closely related reference organism but become consistently less effective when provided orthologous loci from more divergent taxa. While not directly tested here, this result may be caused by the introduction of nontarget reads into gene assembly biased by the reference provided for read-mapping. Although short reads from many taxa may map with high frequency to a highly conserved gene set, short reads originating from paralogous or duplicated genes may contribute to false detection or chimeric sequence construction, depending on the reference-based approach chosen (Salari et al. 2018). Further investigation into this caveat of read-mapping-based phylogenomics is warranted to better characterize best practices.

Benchmarking results against the *Henckelia* dataset demonstrate OrthoGarden's ability to recover accurate topologies in the absence of high-quality assemblies. ROADIES produced a comparable topology to that of other pipelines for the *Anopheles* dataset, represented by high-quality assemblies of multiple mosquitoes, but produced a discordant tree for the *Henckelia* dataset relative to OrthoGarden. *Henceklia* assemblies used in this benchmarking test were generated from short reads using Megahit (Li et al. 2015) for direct comparison with OrthoGarden. Using these fragmented assemblies as input to ROADIES may interfere with its ability to establish orthologous loci or may introduce divergent evolutionary signals between exon and c-gene data.

OrthoGarden produces varying numbers of single copy orthogroups from each dataset, which are processed downstream to create final phylogenies (Figure S16). This observation is likely driven by final assembly quality, annotation, and genomic features of individual taxa. Taxa that are poorly annotated or demonstrate histories of extensive gene duplication are especially predisposed to a low number of recovered single copy orthogroups. This observed variation in the recovered numbers of single copy orthologs demonstrates the value of using an adaptive orthology threshold for final phylogeny construction to maximize sampling of informative loci.

All-vs-all orthology inference, which utilizes loci recovered from all available taxa without a reference gene set, has previously been shown to identify useful orthologs across a wide range of taxa (Emms and Kelly 2019; Lozano-Fernandez 2022; Yang et al. 2023). OrthoGarden's independence from reference orthologs therefore provides a niche among existing tools for conducting phylogenetic inference with nonmodel taxa where a close reference gene set is not available. Further, by using all-vs-all orthology through OrthoFinder (Emms and Kelly 2019) in combination with a modular pipeline approach, input data can include raw sequence read sets, genome assemblies of varying quality, or a combination of both. All-vs-all orthology inference and the taxonomic occupancy threshold implemented to restrict the final orthogroups considered for gene selection protect final topologies from long-branch attraction associated with the introduction of nontarget taxa. Orthology inference further benefits final topologies by excluding contaminant loci. As orthology inference selects for relatively fast-evolving orthologous genes, it is highly unlikely for contaminant taxa to be retained unless they are closely related to in-group taxa. Additional filtering with the optional kraken2 step of OrthoGarden is expected to further reduce the impact of contaminant taxa on final phylogeny outputs.

OrthoGarden's unique supplemental features provide it utility within and outside phylogenomics. Summary tables representing gene and taxon completeness (Table S2) characterize input datasets, allowing users to view their data at a glance and make informed analysis decisions for future runs. For instance, samples producing highly fragmented assemblies can be identified and removed from the pool of included taxa. Intermediate outputs such as draft assemblies and multiple sequence alignments may be similarly applied for other applications.

OrthoGarden is designed to be integrated into a phylogenomics study and not substituted for one entirely. Other approaches used for phylogenetic reconstructions other than maximum likelihood such as coalescent-based methods, Bayesian inference, and others are not included within OrthoGarden at this time. Intermediate outputs from OrthoGarden, such as masked multiple sequence alignments, may be useful as input for these methods of tree construction. A known limitation of orthology inference is its inability to recover accurate phylogenies across evolutionarily distant groups, and OrthoGarden is currently designed for datasets at the order level and below. Future implementations plan to expand to nonexonic and multi-copy loci to handle more distantly diverged datasets. We recommend that results be corroborated with morphological evidence and other tree inference methods to evaluate the efficacy of resulting phylogenies constructed with OrthoGarden.

## Materials and methods

### Pipeline overview

OrthoGarden is an automated pipeline designed for phylogenetic reconstruction via de novo discovery and filtering of orthologous genes from annotated assemblies. Built for Linux systems, OrthoGarden offers users a flexible pipeline designed to accept any combination of taxa in the form of short read Illumina data or pre-assembled genomes. The pipeline, implemented in Nextflow, performs all user-defined downstream processes in a modular and repeatable manner using versioned, containerized tools and custom scripts (Di Tommaso et al. 2017). The pipeline performs a core set of functions with optional steps to increase versatility (Fig. 1). If assembly is required, raw, paired-end read data may be quality checked, filtered and trimmed using FastQC, MultiQC and Cutadapt (Andrews 2010; Martin 2011; Ewels et al. 2016). Optional filtering for nontarget DNA may be performed via Kraken2 (Wood et al. 2019). Reads are assembled using Megahit, a NGS genome assembler chosen for its scalable performance and low resource overhead (Li et al. 2015).

All assembled genomes are then annotated with Augustus using a user-defined training organism (Stanke et al. 2006). Annotated proteins from all input taxa are passed to OrthoFinder to generate a set of orthologous gene groupings (Emms and Kelly 2019). Orthologous genes meeting a defined threshold of percent-included taxa are extracted as nucleotide sequences from OrthoFinder output using custom Python scripts (OrthoFinder Finder). The following steps are applied to produce a cleaned dataset to generate a ML (maximum likelihood) phylogeny from input data. Recovered genes are translated into amino acid sequences and aligned with MAFFT (Katoh and Standley 2013). Corresponding nucleotide sequences are mapped to aligned amino acid sequences to reduce alignment complexity and the impact of synonymous mutations. Resulting alignments may be masked with trimAl at a user-provided threshold (default = 0.4) (Capella-Gutiérrez et al. 2009). A custom Python script may optionally be used to mask third positions to remove the influence of codon heterogeneity (Breinholt and Kawahara 2013; Yang et al. 2023). A maximum likelihood phylogeny is created from resulting alignments with IQ-TREE2 with 1,000 ultrafast bootstraps optimized with nearest neighbor interchange and relaxed hierarchical clustering (Minh et al. 2020). ModelFinder is leveraged to identify the best-performing model for sets of alignments (Kalyaanamoorthy et al. 2017) to minimize the impact of long-branch attraction on the final topology.

Output files for each step of OrthoGarden are created within a directory of the user's choosing and contain subdirectories for each step of the pipeline including documentation on all settings and tools. Previously completed steps may be saved using Nextflow's resume option, making it easy to add or remove taxa and allowing for experimentation in several thresholds defining the final set of orthologous genes.

### Benchmarking OrthoGarden

Benchmarking was conducted to compare phylogenies produced by Orthogarden to those from other comparable phylogenomic tools using identical datasets. OrthoGarden was benchmarked by comparing its performance to existing phylogenomics pipelines using 3 datasets across broad taxonomic groups: Anopheline mosquitoes (Neafsey et al. 2015), yeast (Shen et al. 2018; Dylus et al. 2024), and *Henckelia* plants (Shen et al. 2018). aTRAM 2.0 and read2tree version 0.1.5 were selected for benchmarking as they contain automated steps and represent the most comparable phylogenomic use cases to OrthoGarden. In order to fairly compare the 2 pipelines, aTRAM output was post-processed in a downstream pipeline identical to OrthoGarden. Except when noted, reference orthologs for read-mapping approaches were chosen based on the closest relative to the taxonomic group of each dataset.

### *Anopheles* dataset

The *Anopheles* dataset represents a small set of taxa with a well-documented evolutionary history and abundance of available orthologs from closely related species. The majority of benchmarking tests were conducted with this dataset due to its relatively small size. Paired-end shotgun sequencing reads and assemblies of 21 species for *Anopheles* mosquitoes, including 2 out-groups, were selected for benchmarking (Table S1). A reference topology for accuracy comparisons was obtained from Neafsey et al. (2015) using TreeSnatcher Plus (Laubach et al. 2012). The aTRAM pipeline was run using a reference set of 800 single copy genes present in 90% of taxa from the available *Anopheles* species in OrthoDB (Kriventseva et al. 2019). An additional reference gene set was constructed from orthologs recovered from the more distantly related *Drosophila* using OrthoDB to evaluate how a reference gene set phylogenetically distant from in-group taxa impacts the efficacy of this read-mapping approach. Read2tree was initiated under default parameters, utilizing 3 distinct sets of reference genes acquired from the OMA browser (200 marker genes at 0.9 “Minimum fraction of covered species”) (Altenhoff et al. 2019; Dylus et al. 2024). Taxa retained from OMA reference genes were dropped from final read2tree phylogenies. Marker sets were limited to Anopheles, Drosophila, and Diptera to assess the influence of evolutionary distance in the reference. Two additional read2tree runs for the Diptera group excluding members of Culicidae were performed to assess in-group presence and the impact of gene count (200 and 3,261 marker genes at 0.9 “Minimum fraction of covered species”). OrthoDB and OMA were selected for reference gene creation for aTRAM and read2tree pipelines, respectively, to represent their published best practices. OrthoGarden was benchmarked using default parameters with the same input reads and with Augustus gene training settings of either “anopheles” or “fly.” An OrthoGarden taxa occupancy threshold of 1 for OrthoFinder results was chosen for runs with all assembly inputs, and a threshold of 0.9 was chosen for a run composed only of short read inputs. This taxonomic occupancy threshold was selected to identify phylogenetically relevant genes in a relatively narrow taxonomic group. Both assembly and short read inputs were tested to assess the consistency of results when OrthoGarden is provided different input formats. To this end, 3 different inputs were benchmarked. One with only short read inputs, one with only assembly inputs, and one with mixed input types. ROADIES was run on *Anopheles* dataset assembly inputs under default parameters.

*Anopheles* dataset short read inputs were subset to simulate 1×, 3×, and 5× coverage of the *A. gambiae* genome (264.5 Mb). OrthoGarden was run using these subset short read inputs, and resulting final topologies were assessed for concordance with the appropriate reference tree (Neafsey et al. 2015).

### Saccharomycotina dataset

A large yeast dataset was chosen for benchmarking to evaluate OrthoGarden's performance when supplied an abundance of taxa and genomic information. Assembly-based phylogenomic methods are traditionally constrained by assembly and all-vs-all orthology inference processes that increase computational complexity and analysis time with supplemental taxa. In addition to its scale, this dataset was used for phylogenomic benchmarking by read2tree and has a robust reference phylogeny with a well-studied evolutionary history (Shen et al. 2018; Dylus et al. 2024). The 416 taxa included in the yeast reconstructions produced by Dylus et al. (2024) (Table S2) were selected to create a phylogeny with OrthoGarden under default parameters using the “saccharomyces” Augustus training model and a taxa occupancy threshold of 0.75 for OrthoFinder results. Selected taxa were chosen from Dylus *et al*. to more effectively compare OrthoGarden's performance with read2tree. This threshold value was selected to include informative genes for the wide taxonomic range represented in this dataset. OrthoGarden results are compared to topologies recovered in Shen et al. (2018) and read2tree (Dylus et al. 2024).

### Henckelia dataset

The plant dataset chosen for benchmarking represents a nonmodel group with high evolutionary distance from conventional reference organisms. Existing phylogenetic reconstructions generated from genome and plastome sequences provide a baseline for accuracy. Paired-end shotgun sequencing reads of 29 species of *Henckelia* and out-group taxa (Table S1) were used to recreate the genus phylogeny from Yang et al. (2023). The process of identifying reference genes from a well-annotated in-group reference genome and mapping target loci from target taxa makes this a particularly robust reference topology for benchmarking. The reference topology for accuracy comparisons was recovered from Yang et al. (2023) with TreeSnatcher Plus (Laubach et al. 2012). Accession numbers for reads are available at Table S1. A reference set of 800 single-copy reference genes was created with OrthoDB from *Dorcoceras hygrometricum*, the most closely related organism available (Kriventseva et al. 2019). An aTRAM-based pipeline identical to that leveraged for the *Anopheles* dataset was executed for this dataset (Allen et al. 2018). Read2tree was initiated with a pipeline identical to the read2tree pipeline used for the *Anopheles* dataset, except with a reference gene set derived from *Solanum lycoperiscum* (Dylus et al. 2024). Orthogarden was run under default parameters and a taxa occupancy threshold of 0.75 with selected reads using the *S. lycopersicum* Augustus training model for all taxa, as it is the most closely related Augustus training species available. This threshold was chosen to identify phylogenetically useful genes in a group with high genomic content and structural diversity. ROADIES was run using *Henckelia* dataset assembly intermediate outputs produced from OrthoGarden under default parameters.

### Evaluating performance

Resulting phylogenies were evaluated for accuracy by comparing them with published reference trees. Out-group taxa not represented in ground-truth trees were dropped. JRFD scores were calculated between reference and generated phylogenies with the TreeDist package in RStudio (Smith 2020). Lower JRFD scores represent more concordant topologies between reconstructions. Reference trees were generated from the topologies of phylogenies created in the studies from which the datasets were chosen.

## Supplementary Material

msag053_Supplementary_Data

## Data Availability

Code and documentation for the OrthoGarden pipeline are publicly available through github.com/jacksonhturner/orthogarden. Code used for analyses used in this study can be found at github.com/jacksonhturner/orthogarden_analysis. All reference genome and sequence datasets used to benchmark OrthoGarden are publicly available via NCBI, and specific accession information is available at github.com/jacksonhturner/orthogarden_analysis.

## References

[msag053-B1] Allen JM, LaFrance R, Folk RA, Johnson KP, Guralnick RP. aTRAM 2.0: an improved, flexible locus assembler for NGS data. Evol Bioinforma. 2018:14:1176934318774546. 10.1177/1176934318774546. PMC5987885 29881251

[msag053-B2] Altenhoff AM et al OMA standalone: orthology inference among public and custom genomes and transcriptomes. Genome Res. 2019:29:1152–1163. 10.1101/gr.243212.118. 31235654 PMC6633268

[msag053-B3] Andrews S . 2010. FastQC: A Quality Control Tool for High Throughput Sequence Data [Online]. Available from: http://www.bioinformatics.babraham.ac.uk/projects/fastqc/

[msag053-B4] Breinholt JW, Kawahara AY. Phylotranscriptomics: saturated third Codon positions radically influence the estimation of trees based on next-gen data. Genome Biol Evol. 2013:5:2082–2092. 10.1093/gbe/evt157. 24148944 PMC3845638

[msag053-B5] Cai L, Liu D-T, Zhang P, Dao Z-L. Two new species of Henckelia (Gesneriaceae) from Southeastern Yunnan, China. PhytoKeys. 2019:130:151–160. 10.3897/phytokeys.130.33988. 31534403 PMC6728312

[msag053-B6] Capella-Gutiérrez S, Silla-Martínez JM, Gabaldón T. Trimal: a tool for automated alignment trimming in large-scale phylogenetic analyses. Bioinformatics. 2009:25:1972–1973. 10.1093/bioinformatics/btp348. 19505945 PMC2712344

[msag053-B7] Di Tommaso P et al Nextflow enables reproducible computational workflows. Nat Biotechnol. 2017:35:316–319. 10.1038/nbt.3820. 28398311

[msag053-B8] Dominguez Del Angel V et al Ten steps to get started in genome assembly and annotation. F1000Res. 2018:7:148. 10.12688/f1000research.13598.1. PMC5850084 29568489

[msag053-B9] Dylus D, Altenhoff A, Majidian S, Sedlazeck FJ, Dessimoz C. Inference of phylogenetic trees directly from raw sequencing reads using Read2Tree. Nat Biotechnol. 2024:42:139–147. 10.1038/s41587-023-01753-4. 37081138 PMC10791578

[msag053-B10] Emms DM, Kelly S. OrthoFinder: phylogenetic orthology inference for comparative genomics. Genome Biol. 2019:20:238. 10.1186/s13059-019-1832-y. 31727128 PMC6857279

[msag053-B11] Ewels P, Magnusson M, Lundin S, Käller M. MultiQC: summarize analysis results for multiple tools and samples in a single report. Bioinformatics. 2016:32:3047–3048. 10.1093/bioinformatics/btw354. 27312411 PMC5039924

[msag053-B12] Fleming JF, Valero-Gracia A, Struck TH. Identifying and addressing methodological incongruence in phylogenomics: a review. Evol Appl. 2023:16:1087–1104. 10.1111/eva.13565. 37360032 PMC10286231

[msag053-B13] Gupta A, Mirarab S, Turakhia Y. Accurate, scalable, and fully automated inference of species trees from raw genome assemblies using ROADIES. Proc Natl Acad Sci U S A. 2025:122:e2500553122. 10.1073/pnas.2500553122. 40314967 PMC12088440

[msag053-B14] Han S et al Local assembly of long reads enables phylogenomics of transposable elements in a polyploid cell line. Nucleic Acids Res. 2022:50:e124. 10.1093/nar/gkac794. 36156149 PMC9757076

[msag053-B15] Johnson KP et al Phylogenomics and the evolution of hemipteroid insects. Proc Natl Acad Sci U S A. 2018:115:12775–12780. 10.1073/pnas.1815820115. 30478043 PMC6294958

[msag053-B16] Kalyaanamoorthy S et al ModelFinder: fast model selection for accurate phylogenetic estimates. Nat Methods. 2017:14:587–589. 10.1038/nmeth.4285. 28481363 PMC5453245

[msag053-B17] Katoh K, Standley DM. MAFFT multiple sequence alignment software version 7: improvements in performance and usability. Mol Biol Evol. 2013:30:772–780. 10.1093/molbev/mst010. 23329690 PMC3603318

[msag053-B18] Kriventseva EV et al OrthoDB v10: sampling the diversity of animal, plant, fungal, protist, bacterial and viral genomes for evolutionary and functional annotations of orthologs. Nucleic Acids Res. 2019:47:D807–D811. 10.1093/nar/gky1053. 30395283 PMC6323947

[msag053-B19] Laubach T, Von Haeseler A, Lercher MJ. TreeSnatcher plus: capturing phylogenetic trees from images. BMC Bioinformatics. 2012:13:110. 10.1186/1471-2105-13-110. 22624611 PMC3411374

[msag053-B20] Li D, Liu C-M, Luo R, Sadakane K, Lam T-W. MEGAHIT: an ultra-fast single-node solution for large and complex metagenomics assembly via succinct *de Bruijn* graph. Bioinformatics. 2015:31:1674–1676. 10.1093/bioinformatics/btv033. 25609793

[msag053-B21] Lozano-Fernandez J . A practical guide to design and assess a phylogenomic study. Genome Biol Evol. 2022:14:evac129. 10.1093/gbe/evac129. 35946263 PMC9452790

[msag053-B22] Martin M . Cutadapt removes adapter sequences from high-throughput sequencing reads. EMBnet J. 2011:17:10. 10.14806/ej.17.1.200.

[msag053-B23] Minh BQ et al IQ-TREE 2: new models and efficient methods for phylogenetic inference in the genomic era. Mol Biol Evol. 2020:37:1530–1534. 10.1093/molbev/msaa015. 32011700 PMC7182206

[msag053-B24] Montoliu-Nerin M et al In-depth phylogenomic analysis of arbuscular mycorrhizal fungi based on a comprehensive set of de novo genome assemblies. Front Fungal Biol. 2021:2:716385. 10.3389/ffunb.2021.716385. 37744125 PMC10512289

[msag053-B25] Neafsey DE et al Highly evolvable malaria vectors: the genomes of 16 *Anopheles* mosquitoes. Science. 2015:347:1258522. 10.1126/science.1258522. 25554792 PMC4380271

[msag053-B26] Salari F, Zare-Mirakabad F, Sadeghi M, Rokni-Zadeh H. Assessing the impact of exact reads on reducing the error rate of read mapping. BMC Bioinformatics. 2018:19:406. 10.1186/s12859-018-2432-7. 30400807 PMC6220446

[msag053-B27] Shen X-X et al Tempo and mode of genome evolution in the budding yeast subphylum. Cell. 2018:175:1533–1545.e20. 10.1016/j.cell.2018.10.023. 30415838 PMC6291210

[msag053-B28] Smith MR . Information theoretic generalized Robinson–Foulds metrics for comparing phylogenetic trees. Bioinformatics. 2020:36:5007–5013. 10.1093/bioinformatics/btaa614. 32619004

[msag053-B29] Stanke M et al AUGUSTUS: ab initio prediction of alternative transcripts. Nucleic Acids Res. 2006:34:W435–W439. 10.1093/nar/gkl200. 16845043 PMC1538822

[msag053-B30] Steenwyk JL, Li Y, Zhou X, Shen X-X, Rokas A. Incongruence in the phylogenomics era. Nat Rev Genet. 2023:24:834–850. 10.1038/s41576-023-00620-x. 37369847 PMC11499941

[msag053-B31] Stiller J et al Complexity of avian evolution revealed by family-level genomes. Nature. 2024:629:851–860. 10.1038/s41586-024-07323-1. 38560995 PMC11111414

[msag053-B32] Tice AK et al PhyloFisher: a phylogenomic package for resolving eukaryotic relationships. Hydrobiol. 2021:4:19. 10.1371/journal.pbio.3001365. PMC8345874 34358228

[msag053-B33] Wiens JJ . The role of morphological data in phylogeny reconstruction. Syst Biol. 2004:53:653–661. 10.1080/10635150490472959. 15371253

[msag053-B34] Wood DE, Lu J, Langmead B. Improved metagenomic analysis with Kraken 2. Genome Biol. 2019:20:257. 10.1186/s13059-019-1891-0. 31779668 PMC6883579

[msag053-B35] Yang L-H, Shi X-Z, Wen F, Kang M. Phylogenomics reveals widespread hybridization and polyploidization in *Henckelia* (Gesneriaceae). Ann Bot. 2023:131:953–966. 10.1093/aob/mcad047. 37177810 PMC10332401

[msag053-B36] Young AD, Gillung JP. Phylogenomics—principles, opportunities and pitfalls of big-data phylogenetics. Syst Entomol. 2020:45:225–247. 10.1111/syen.12406.

[msag053-B37] Zuntini AR et al Phylogenomics and the rise of the angiosperms. Nature. 2024:629:843–850. 10.1038/s41586-024-07324-0. 38658746 PMC11111409

