# Supplementary material for: OrthoGarden: a pipeline for propagating phylogenetic trees for nonmodel organisms from short reads and de novo genome assemblies
Source: Mol Biol Evol. 2026 Feb 27;43(3):msag053. doi: 10.1093/molbev/msag053 (PMC12996765; doi:10.1093/molbev/msag053)
Supplement: msag053_Supplementary_Data [file msag053_supplementary_data.zip › OG_Supplementary_Figures_1_through_11.pdf]

This supplementary file contains final benchmarking phylogenies used in Figures 2 and 4 of *OrthoGarden: a pipeline for propagating phylogenetic trees for non-model organisms from short reads and de novo genome assemblies*. Further details concerning the creation of these figures are available in the "Implementation" section of this manuscript.

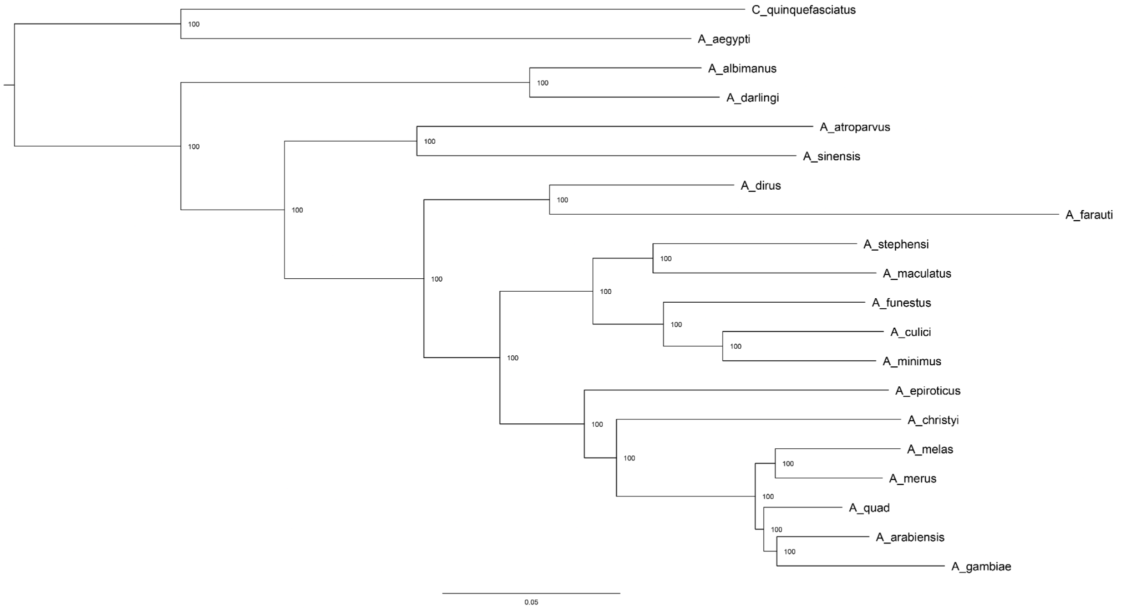

Supplementary Figure 1: Final phylogeny created with aTRAM using the *Anopheles* dataset and an *Anopheles* reference acquired through OrthoDB.

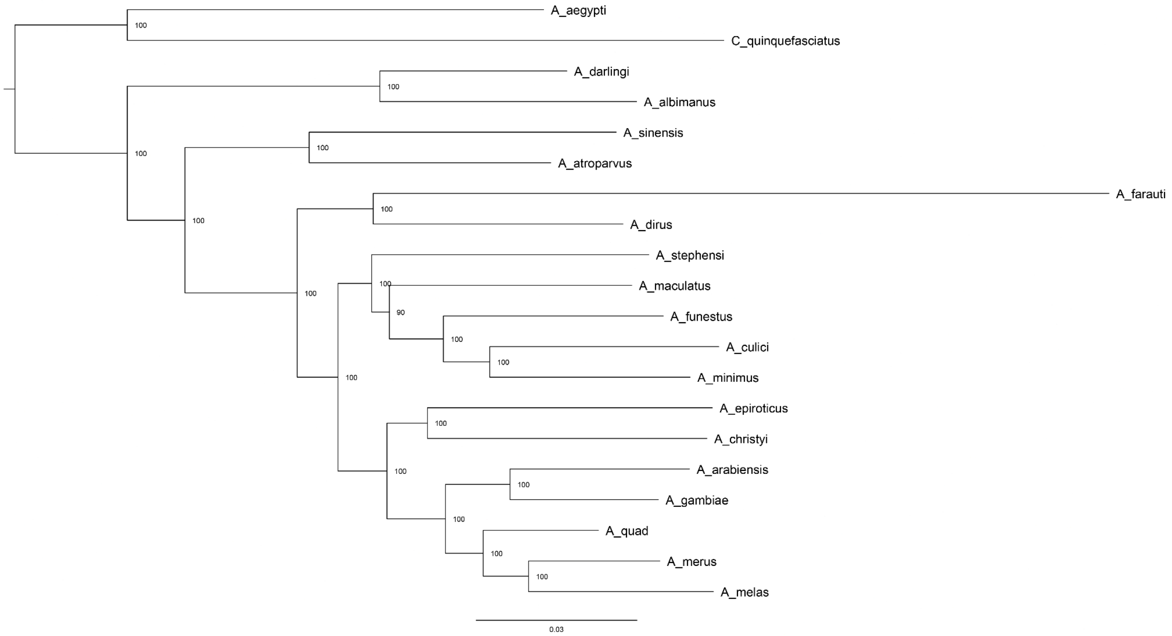

Supplementary Figure 2: Final phylogeny created with aTRAM using the *Anopheles* dataset and a *Drosophila* reference acquired through OrthoDB.

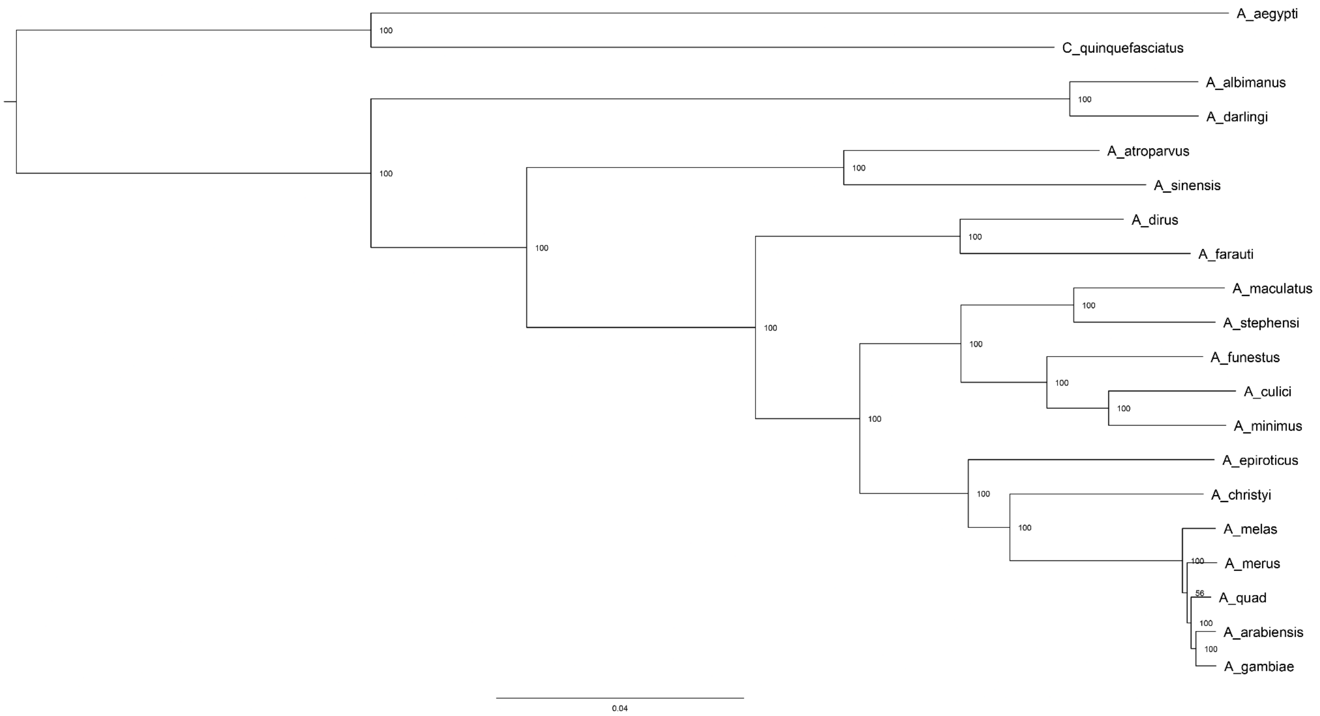

Supplementary Figure 3: Final phylogeny created with OrthoGarden using the *Anopheles* dataset with assembly inputs.

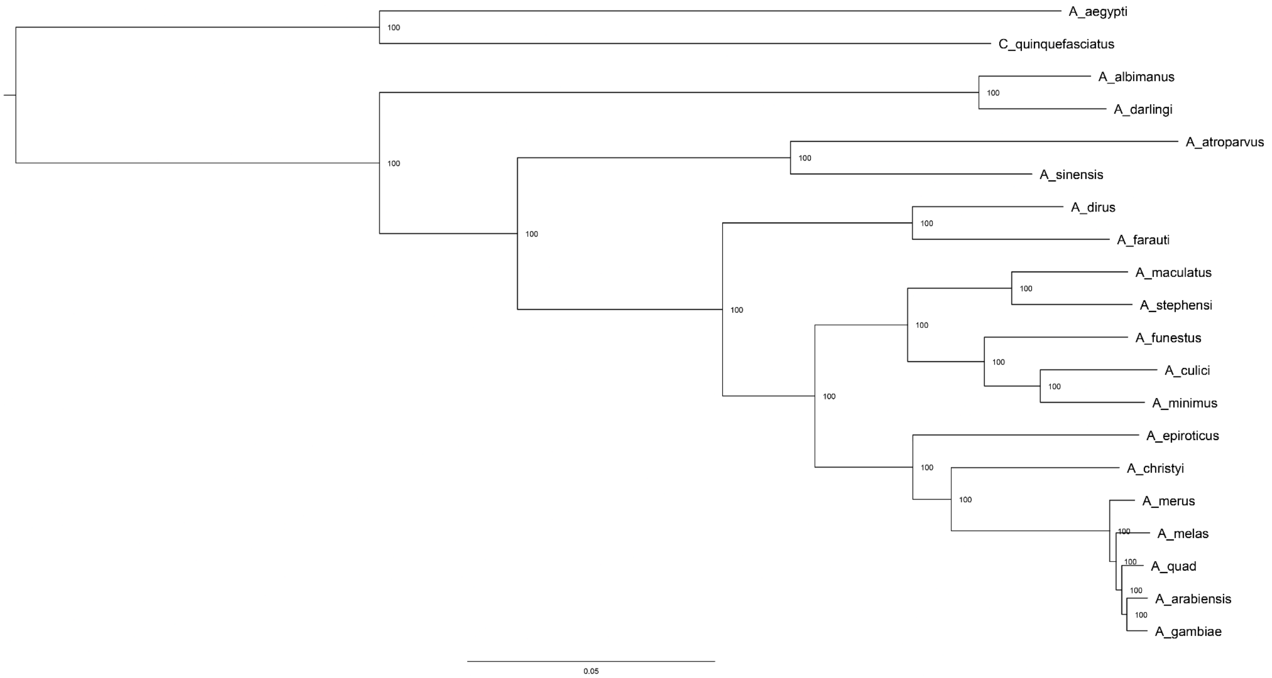

Supplementary Figure 4: Final phylogeny created with OrthoGarden using the *Anopheles* dataset with short-read inputs.

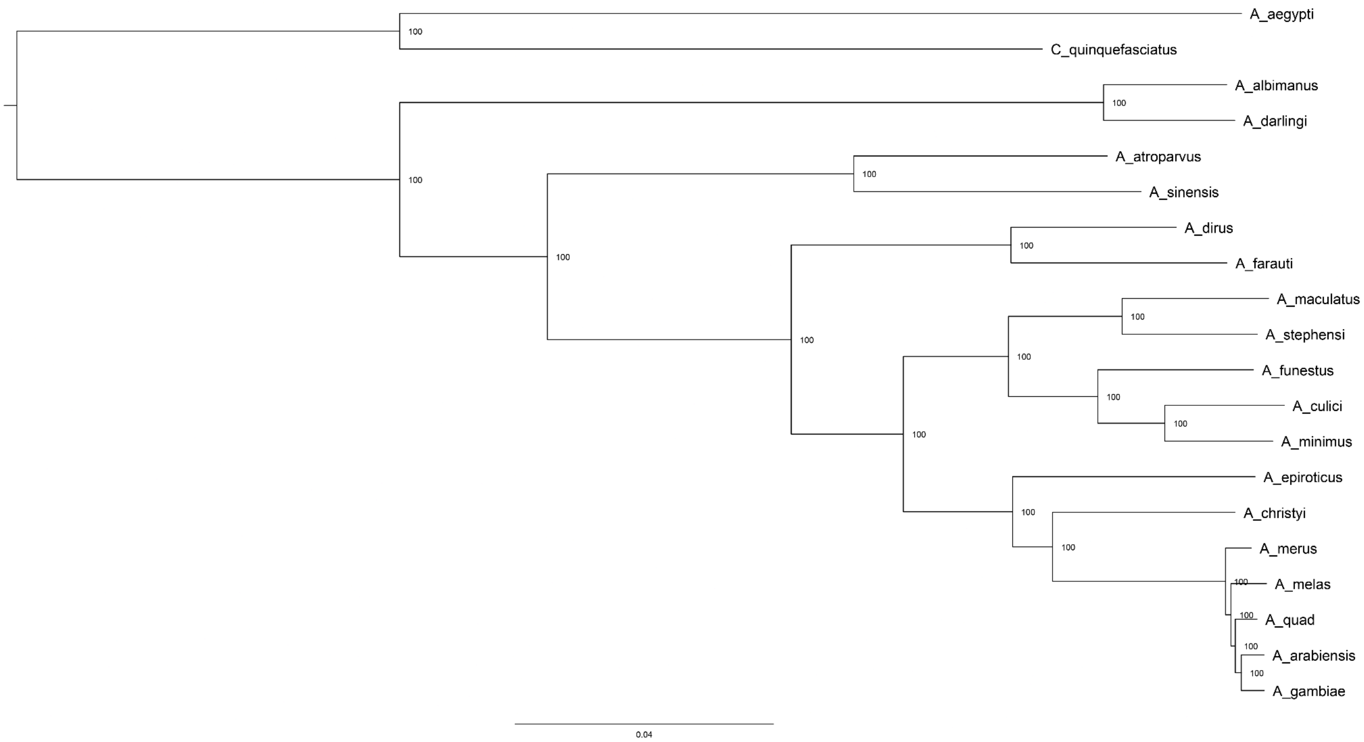

Supplementary Figure 5: Final phylogeny created with OrthoGarden using the *Anopheles* dataset with short-read inputs and the "fly" augustus reference.

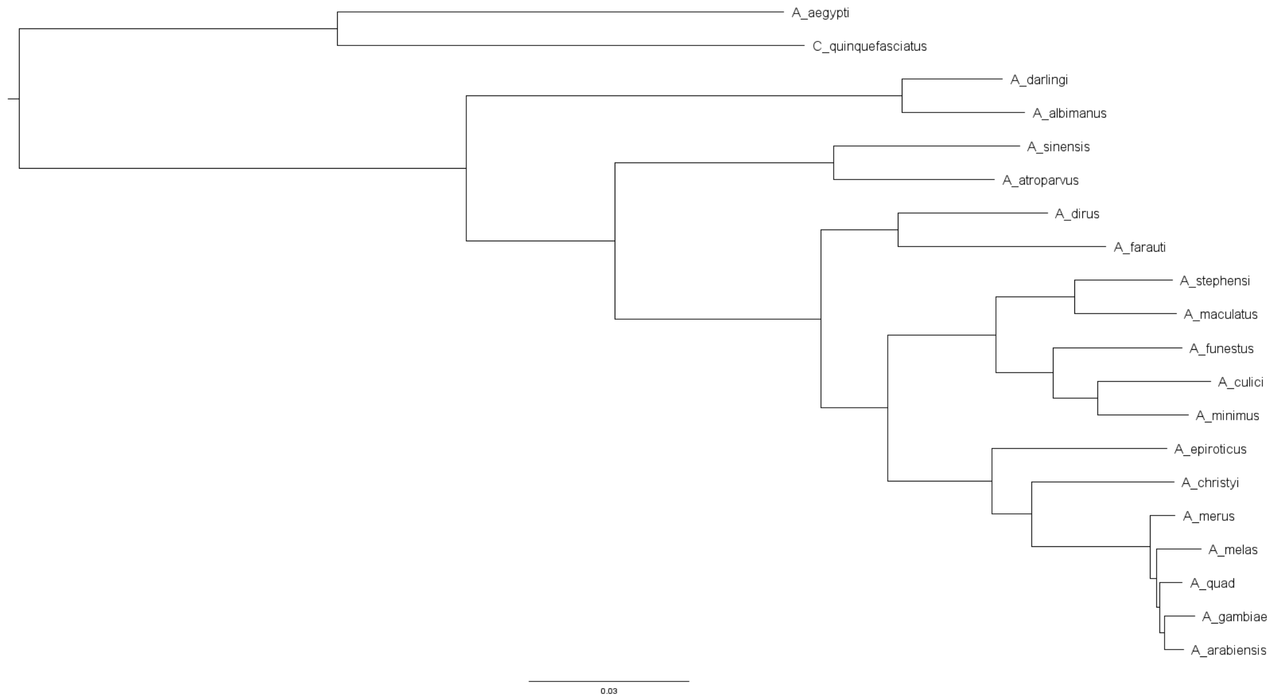

Supplementary Figure 6: Final phylogeny created with read2tree using the *Anopheles* dataset with Diptera OMA genes.

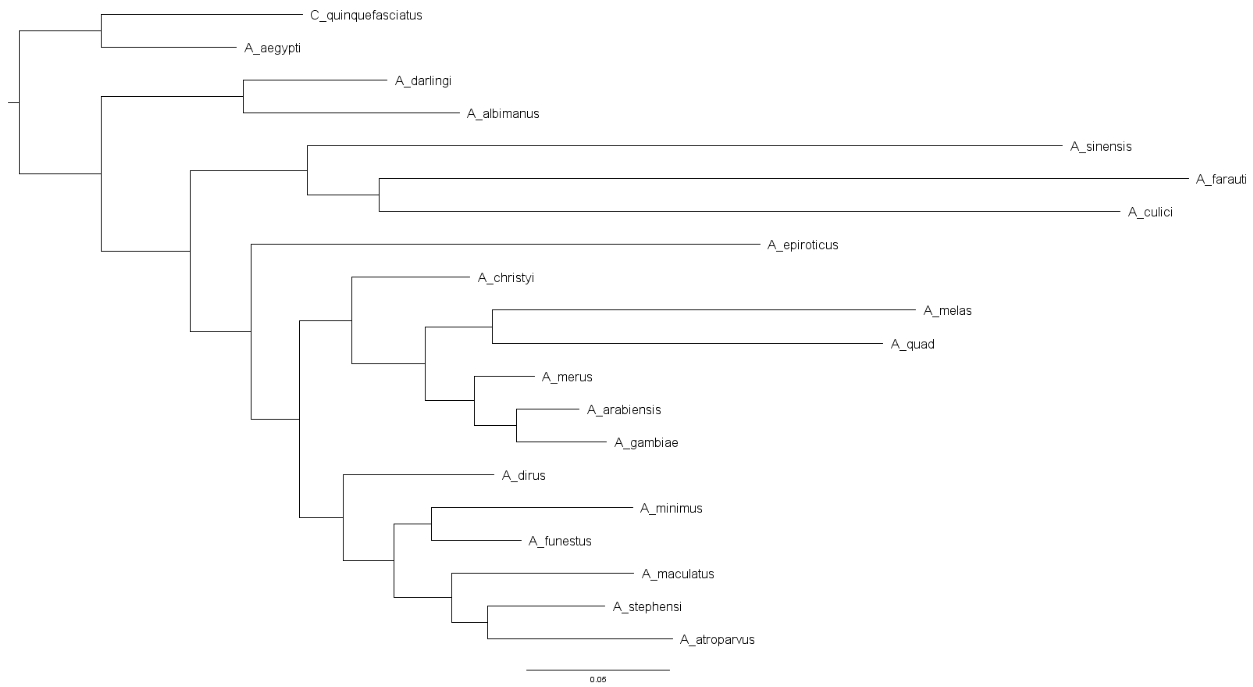

Supplementary Figure 7: Final phylogeny created with read2tree using the *Anopheles* dataset with Drosophila OMA genes.

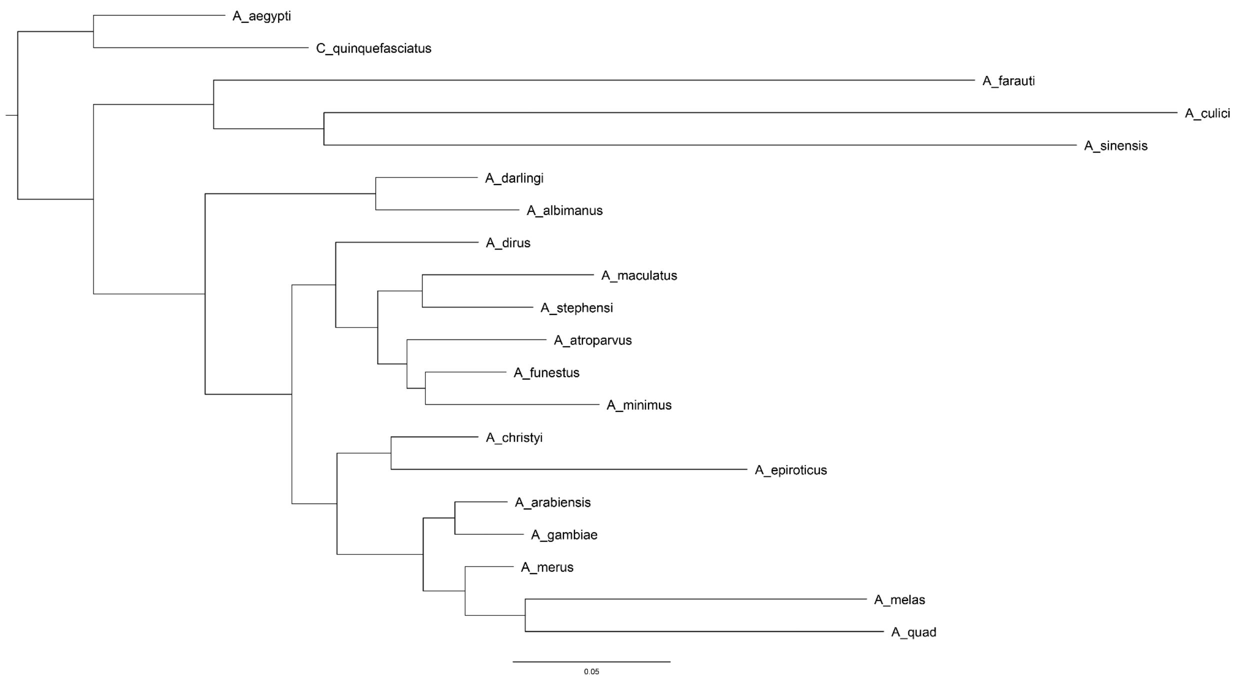

Supplementary Figure 8: Final phylogeny created with read2tree using the *Anopheles* dataset with Diptera OMA genes with *Anopheles* genes excluded.

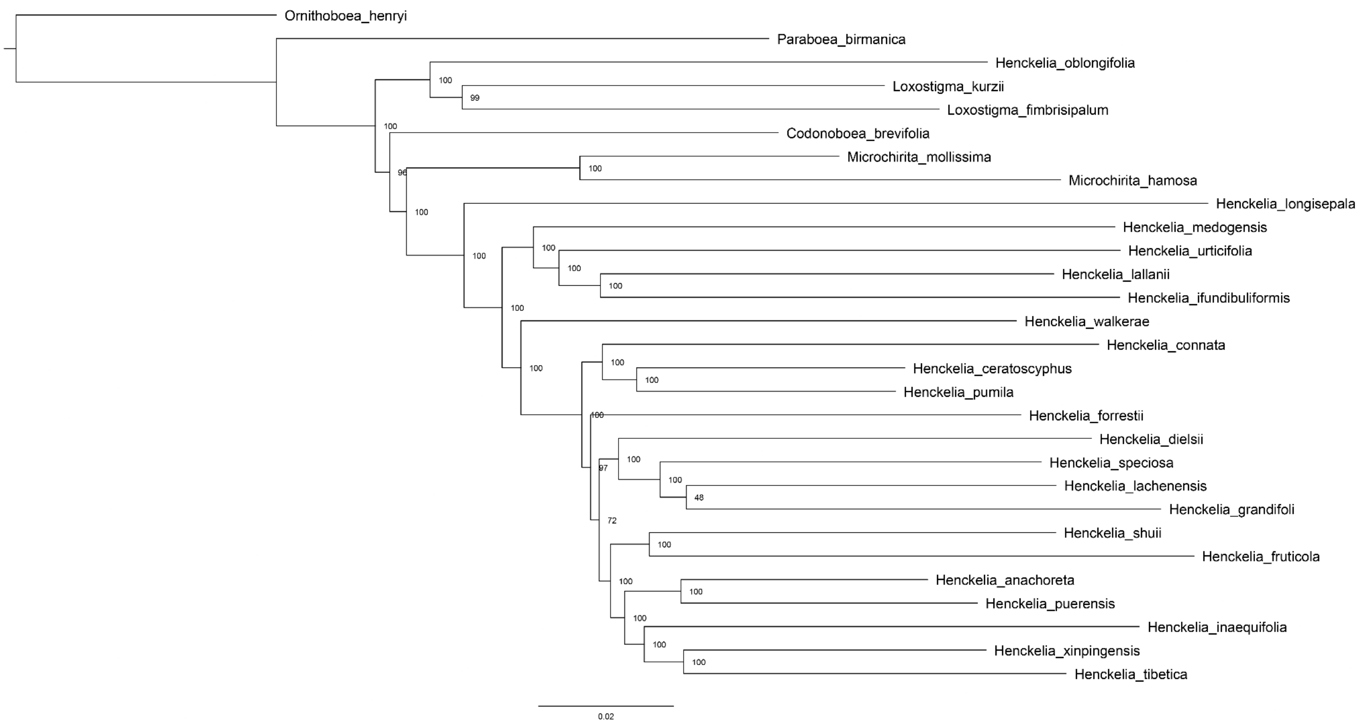

Supplementary Figure 9: Final phylogeny created with aTRAM using the *Henckelia* dataset.

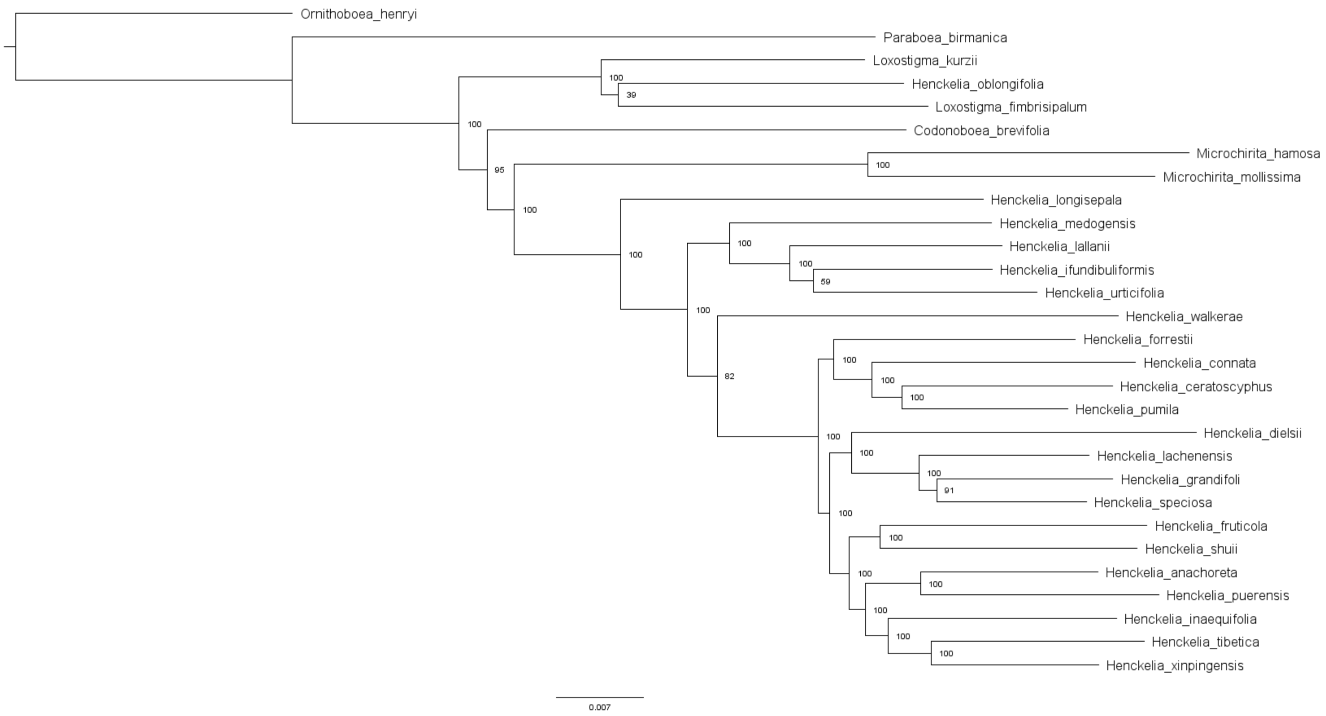

Supplementary Figure 10: Final phylogeny created with OrthoGarden using the *Henckelia* dataset.

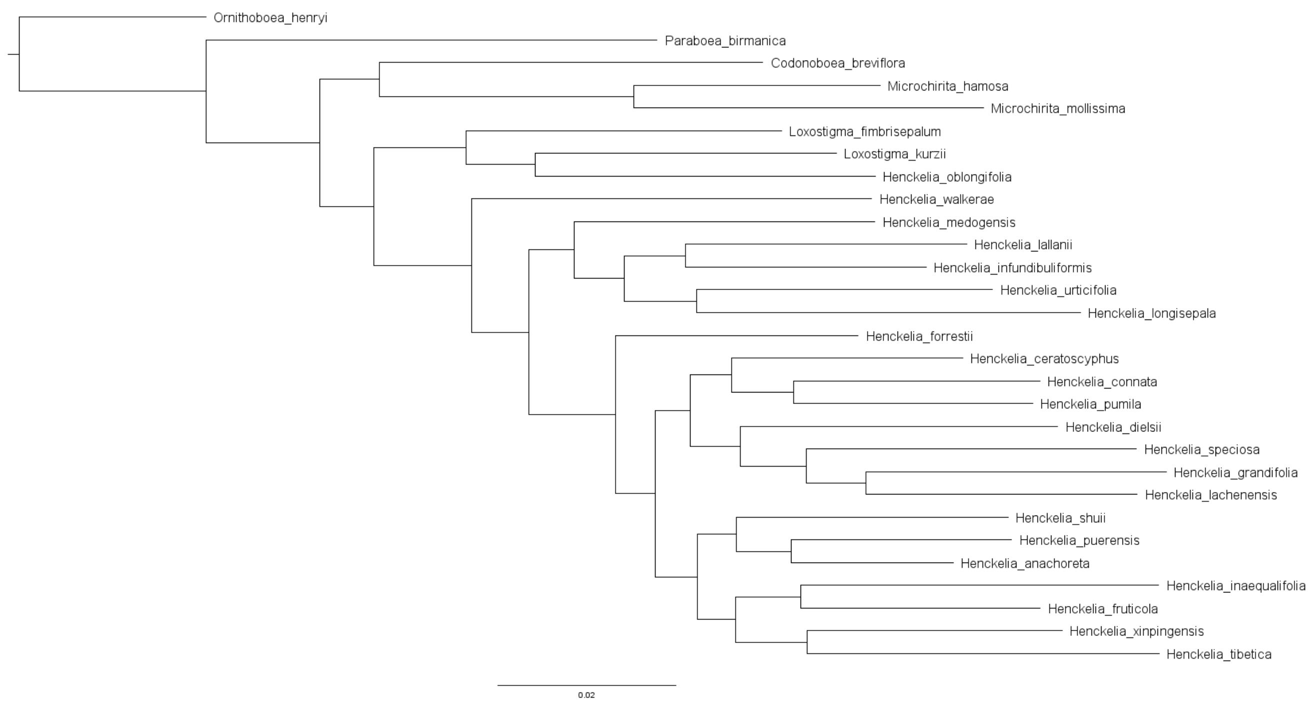

Supplementary Figure 11: Final phylogeny created with read2tree using the *Henckelia* dataset.
