# Supplementary material for: OrthoGarden: a pipeline for propagating phylogenetic trees for nonmodel organisms from short reads and de novo genome assemblies
Source: Mol Biol Evol. 2026 Feb 27;43(3):msag053. doi: 10.1093/molbev/msag053 (PMC12996765; doi:10.1093/molbev/msag053)
Supplement: msag053_Supplementary_Data [file msag053_supplementary_data.zip › OG_Supplementary_Figure_15_Anopheles_coverage.pdf]

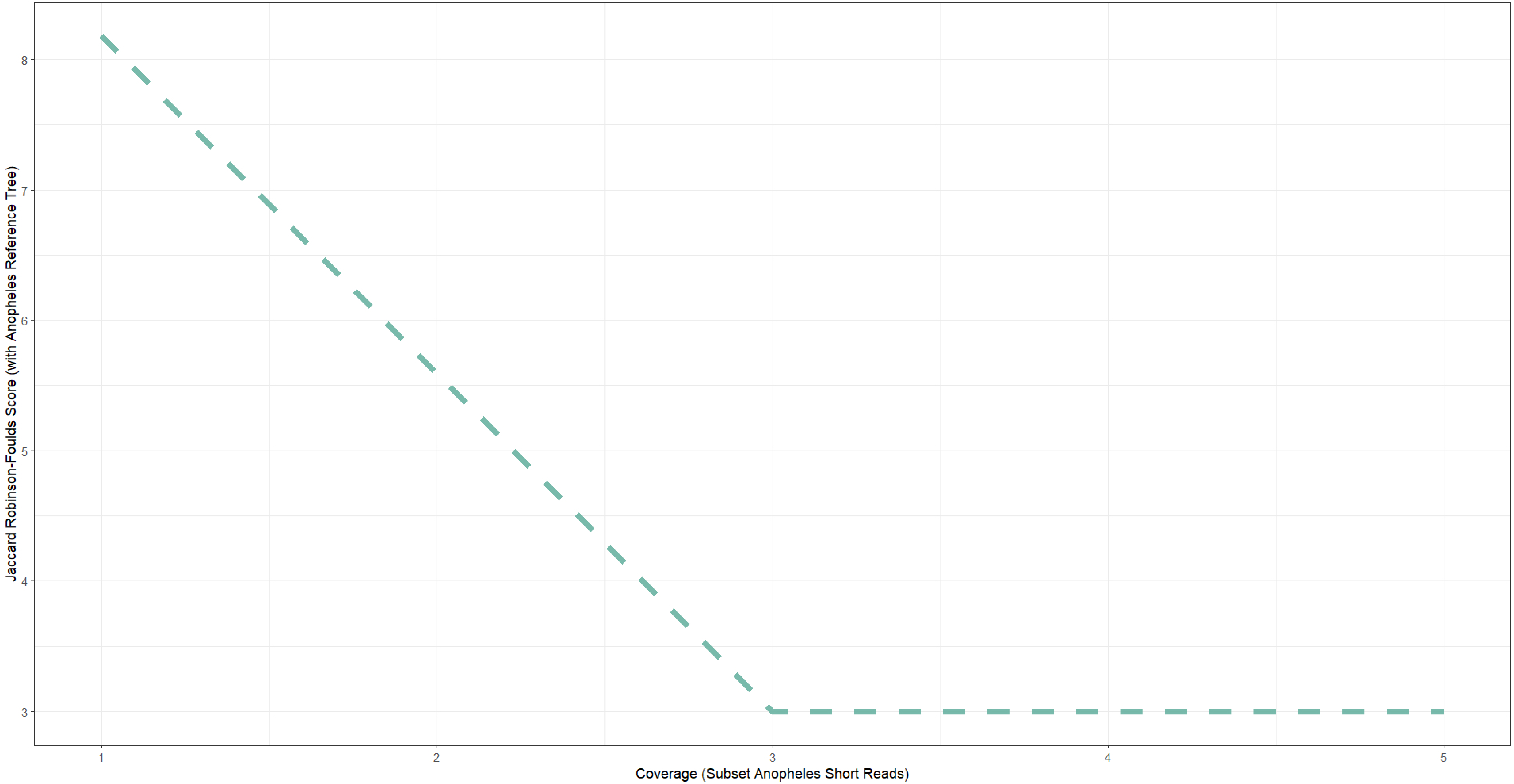

Supplementary Fig. 15. Benchmarking for OrthoGarden using *Anopheles* short reads subset at 1x, 3x, and 5x coverage of *A. gambiae*. Jaccard Robinson-Foulds distances for each benchmarking test are used to measure concordance with a reference phylogeny for the *Anopheles* dataset (Neafsey et al. 2015).
