# Supplementary material for: OrthoGarden: a pipeline for propagating phylogenetic trees for nonmodel organisms from short reads and de novo genome assemblies
Source: Mol Biol Evol. 2026 Feb 27;43(3):msag053. doi: 10.1093/molbev/msag053 (PMC12996765; doi:10.1093/molbev/msag053)
Supplement: msag053_Supplementary_Data [file msag053_supplementary_data.zip › OG_Supplementary_Figure_16_single_copy_orthogroups.pdf]

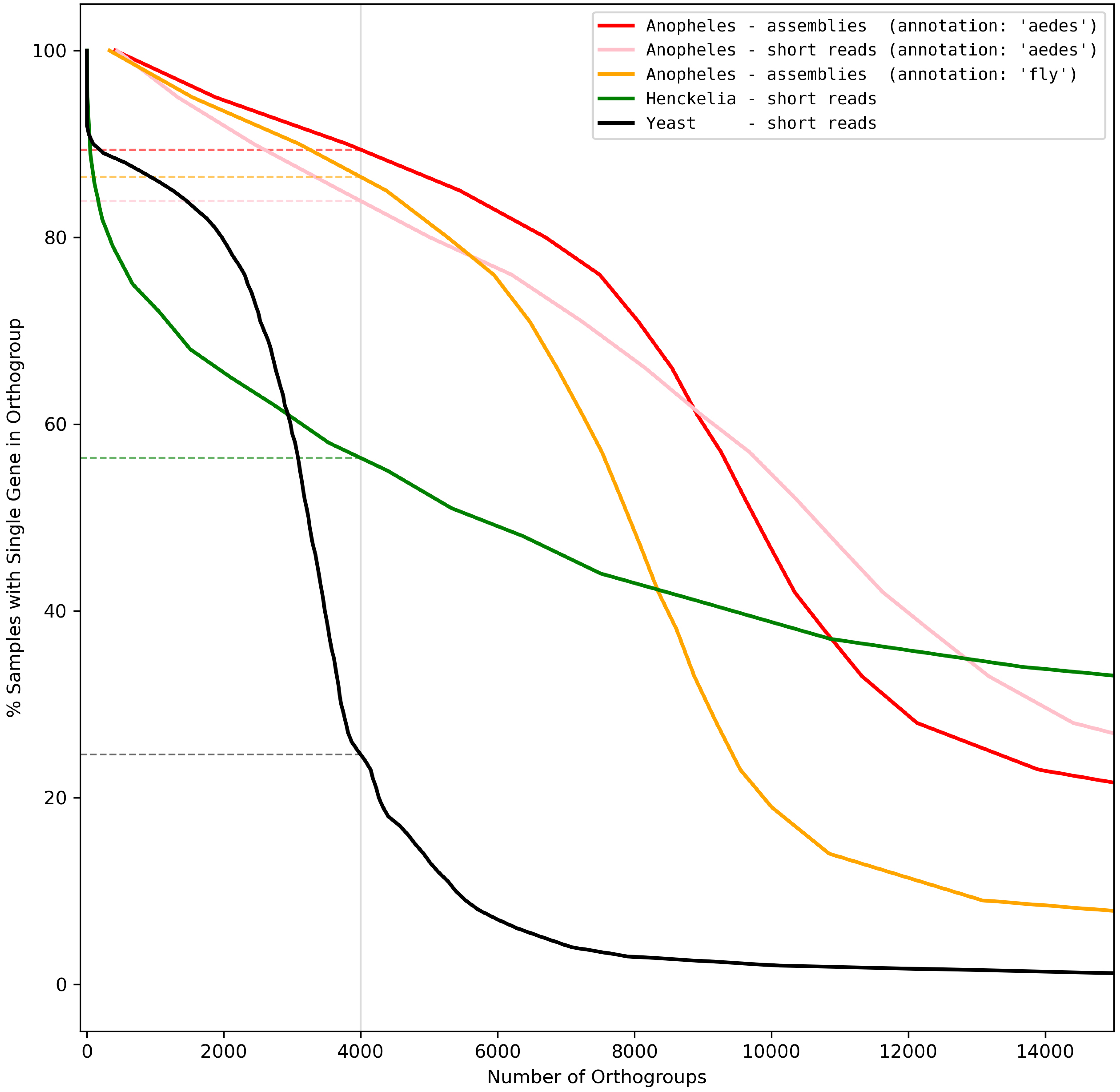


**Supplementary Fig.15.** Relationship between number of recovered orthogrouns and percent occurence of single genes in orthogroup across OrthoGarden benchmarking runs. The percent of single copy genes in each run for 4000 genes is displayed as dashed horizontal lines.
