# Supplementary figures and images for: OrthoGarden: a pipeline for propagating phylogenetic trees for nonmodel organisms from short reads and de novo genome assemblies
Source: Mol Biol Evol. 2026 Feb 27;43(3):msag053. doi: 10.1093/molbev/msag053 (PMC12996765; doi:10.1093/molbev/msag053)

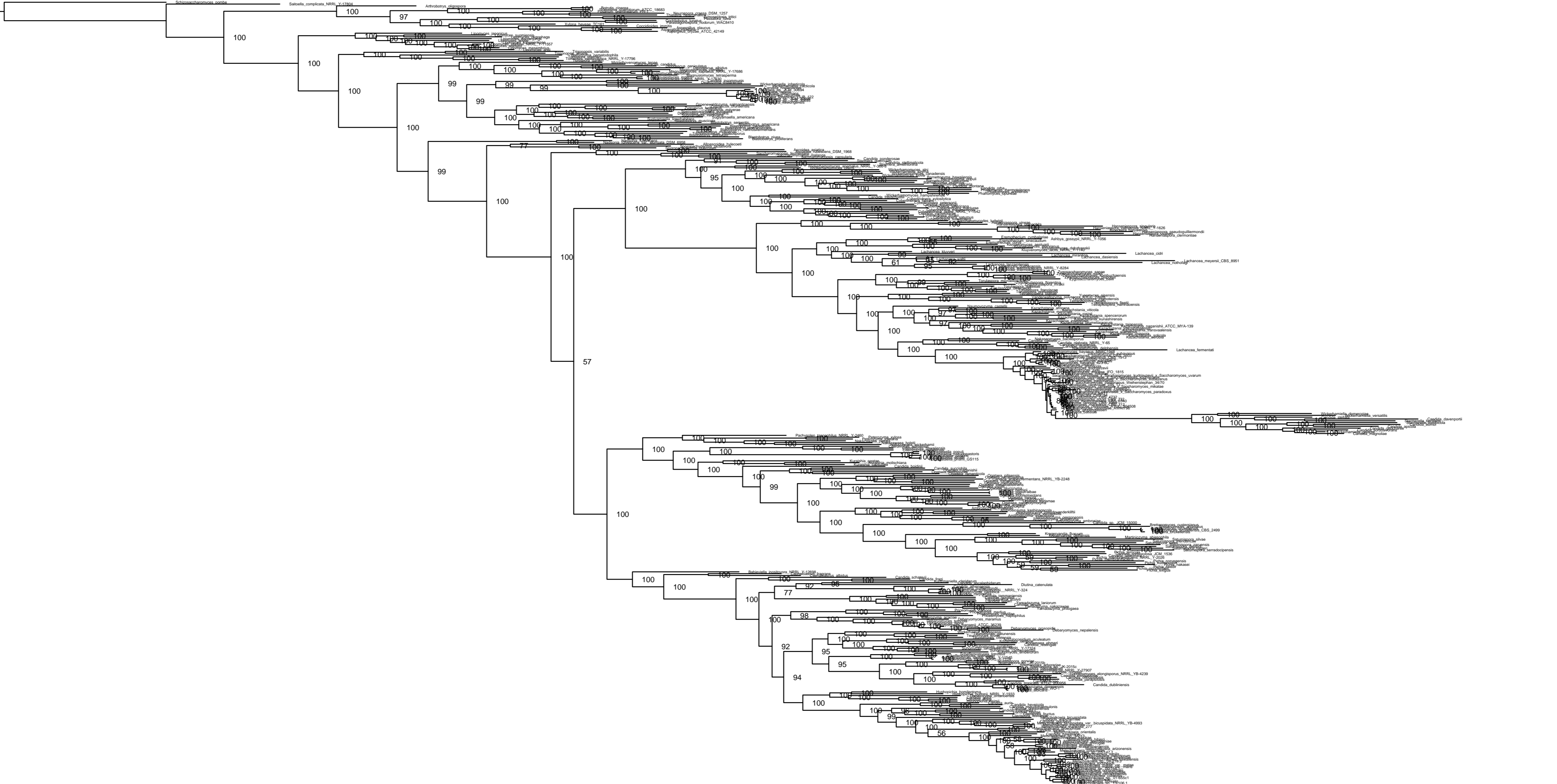

Supplement: msag053_Supplementary_Data [file msag053_supplementary_data.zip › OG_Supplementary_Figure_13_r2t_yeast.pdf]

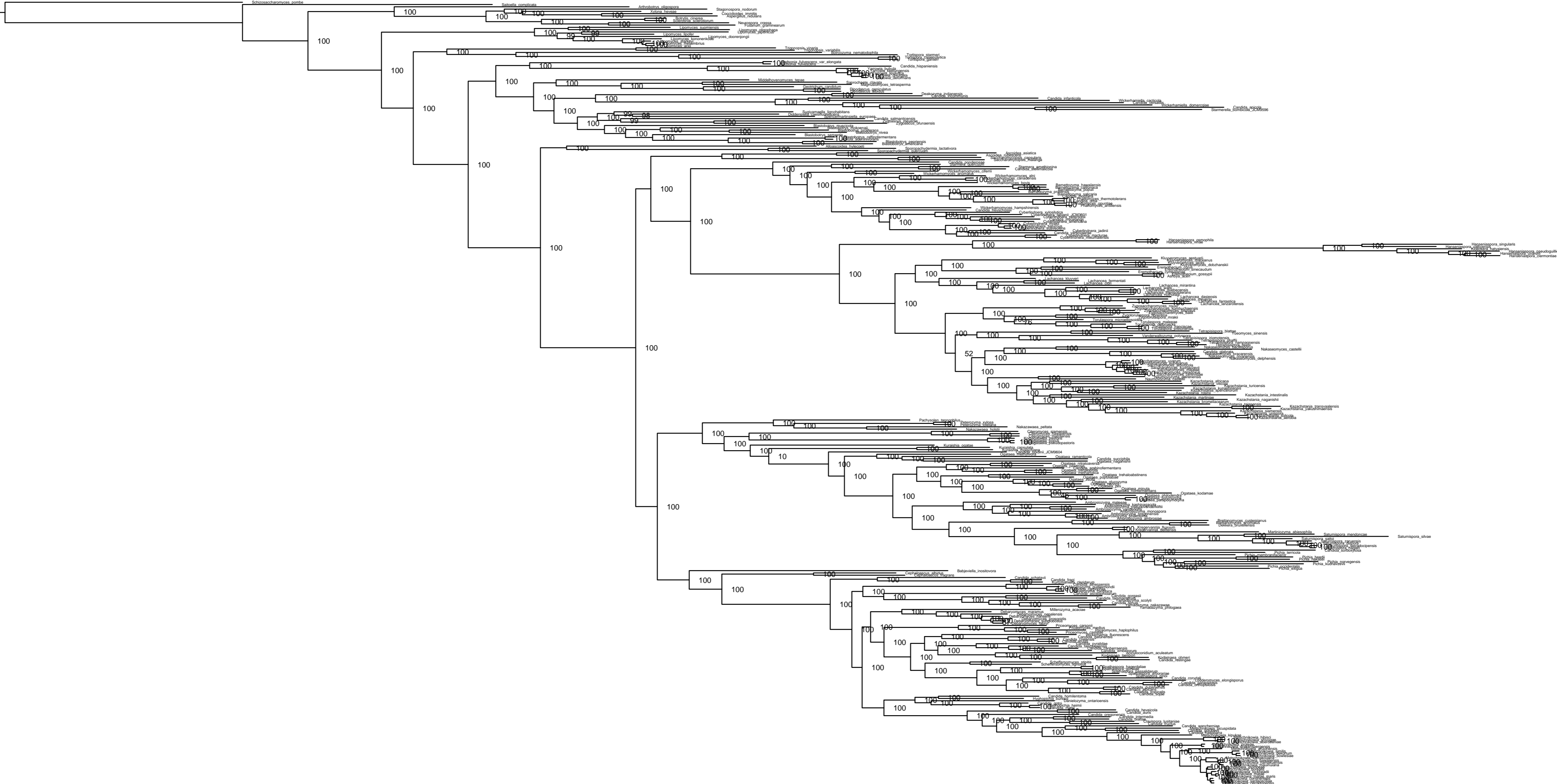

Supplement: msag053_Supplementary_Data [file msag053_supplementary_data.zip › OG_Supplementary_Figure_14_Shen_et_al_yeast.pdf]
