# Supplementary material for: OrthoGarden: a pipeline for propagating phylogenetic trees for nonmodel organisms from short reads and de novo genome assemblies
Source: Mol Biol Evol. 2026 Feb 27;43(3):msag053. doi: 10.1093/molbev/msag053 (PMC12996765; doi:10.1093/molbev/msag053)
Supplement: msag053_Supplementary_Data [file OG_Supplementary_Figure_12_OG_yeast.pdf › OG_Supplementary_Figure_12_OG_yeast.pdf]

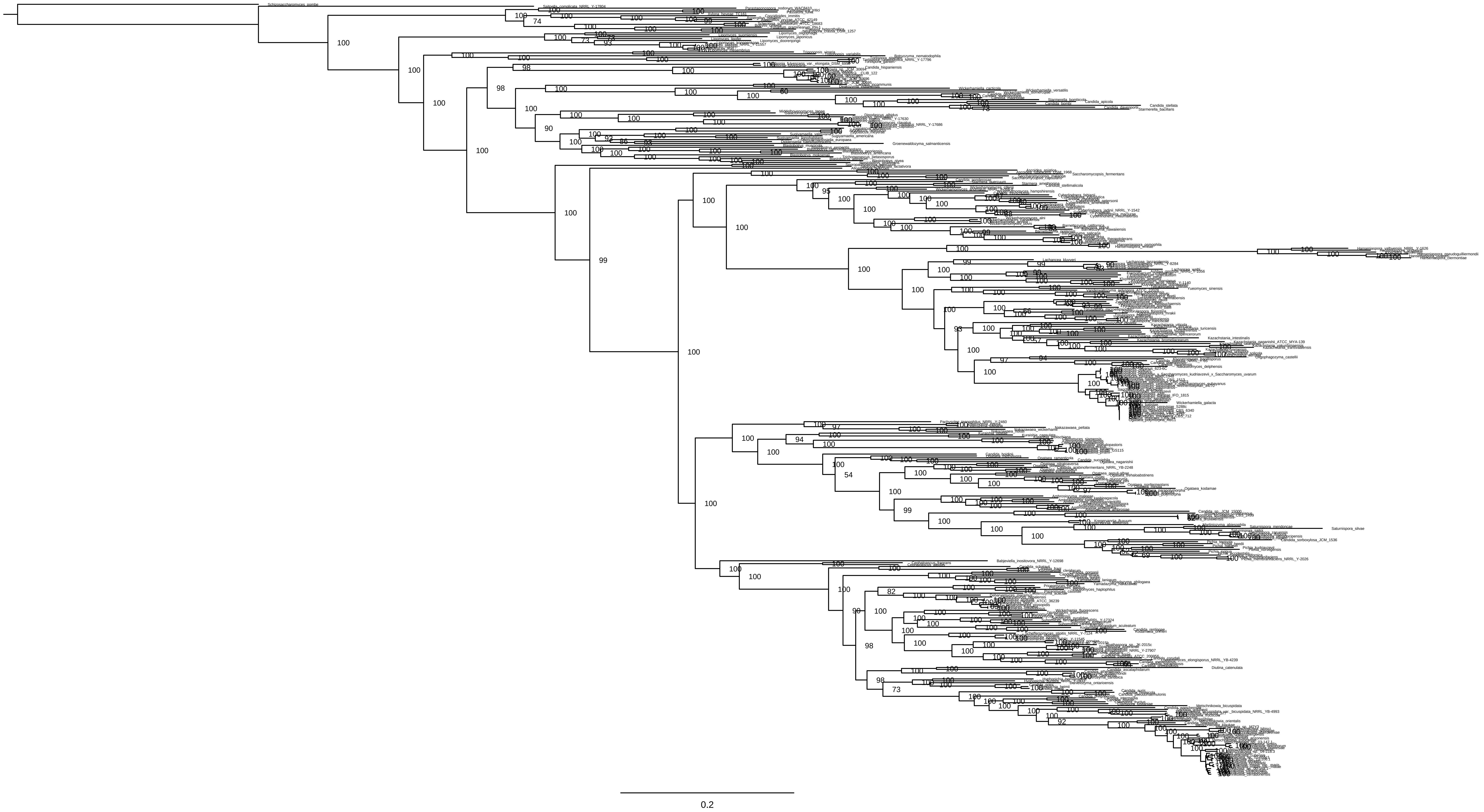
0.2
